# Oak Bark Allometry and Fire Survival Strategies in the Chihuahuan Desert Sky Islands, Texas, USA

**DOI:** 10.1371/journal.pone.0079285

**Published:** 2013-11-14

**Authors:** Dylan W. Schwilk, Maria S. Gaetani, Helen M. Poulos

**Affiliations:** 1 Department of Biological Sciences, Texas Tech University, Lubbock, Texas, United States of America; 2 College of the Environment, Wesleyan University, Middletown, Connecticut, United States of America; The Ohio State University, United States of America

## Abstract

Trees may survive fire through persistence of above or below ground structures. Investment in bark aids in above-ground survival while investment in carbohydrate storage aids in recovery through resprouting and is especially important following above-ground tissue loss. We investigated bark allocation and carbohydrate investment in eight common oak (*Quercus*) species of Sky Island mountain ranges in west Texas. We hypothesized that relative investment in bark and carbohydrates changes with tree age and with fire regime: We predicted delayed investment in bark (positive allometry) and early investment in carbohydrates (negative allometry) under lower frequency, high severity fire regimes found in wetter microclimates. Common oaks of the Texas Trans-Pecos region (*Quercus emoryi*, *Q. gambelii*, *Q. gravesii*, *Q. grisea*, *Q. hypoleucoides*, *Q. muehlenbergii*, and *Q. pungens*) were sampled in three mountain ranges with historically mixed fire regimes: the Chisos Mountains, the Davis Mountains and the Guadalupe Mountains. Bark thickness was measured on individuals representing the full span of sizes found. Carbohydrate concentration in taproots was measured after initial leaf flush. Bark thickness was compared to bole diameter and allometries were analyzed using major axis regression on log-transformed measurements. We found that bark allocation strategies varied among species that can co-occur but have different habitat preferences. Investment patterns in bark were related to soil moisture preference and drought tolerance and, by proxy, to expected fire regime. Dry site species had shallower allometries with allometric coefficients ranging from less than one (negative allometry) to near one (isometric investment). Wet site species, on the other hand, had larger allometric coefficients, indicating delayed investment to defense. Contrary to our expectation, root carbohydrate concentrations were similar across all species and sizes, suggesting that any differences in below ground storage are likely to be in total volume of storage tissue rather than in carbohydrate concentration.

## Introduction

Fire has been a powerful disturbance on the global landscape for hundreds of thousands of years, promoting traits in plants which confer an advantage in the presence of fire [1], [2]. Understanding variation in fire response strategies both across and within fire regimes is a major goal of plant fire ecology [1], [3]–[5]. There are two broad classes of traits that allow populations to persist in the face of recurring fire: those that increase individual survival and those that facilitate post fire seedling recruitment and establishment [3], [6]. Many previous studies have focused on this dichotomy by contrasting non sprouting fire recruiters with basal resprouters [6]–[8] or with fire-surviving species that rely on height and/or bark to avoid tissue damage [9], [10]. Although such work has often treated persistence (especially resprouting) as a binary trait, there has been increasing evidence of the importance of resprouting across diverse ecosystems and recognition of the importance of variability among resprouting responses [11]–[13].

Adult plant survival can provide an obvious advantage to plants in disturbance prone habitats, but such survival may require investment in defense. Plants can survive fire by protecting above ground tissue from heat damage or by relying on basal or below ground resprouting after above ground tissue is killed [3], [14]. For trees, thick bark and tall height that keeps leaves and young tissue away from the flames of a surface fire are the main traits associated with above ground survival [15], [16], while basal or below ground resprouting can allow individual survival even when above ground tissue is killed. Even plants that lose all above ground tissue during a fire and resprout basally may have an advantage over the seedlings of non-sprouting post-fire recruiters because the resprout has access to stored carbon and a more extensive root system than the seedling.

For aerially resprouting species (from epicormic, stem or apical buds), insulating bark can play an important role in protecting stems and epicormic buds even in cases where there is extensive canopy heating and tissue loss [10], [13], [17]–[19]. Thick bark protects a plant's cambium and buds from heating over 60°C, the temperature generally associated with tissue mortality [10], [20], [21]. Although fire ecologists have long characterized tree species as having thick or thin bark [22]–[24], bark thickness as a proportion of stem size changes through the life of individual plants. Empirical and theoretical work has suggested that relative bark thickness may be more important than absolute thickness; differing habitats and fire regimes should lead to different amounts of total investment in bark as well as differences in the timing of investment through a tree's life [18], [25]–[27].

To our knowledge, only one previous paper has explicitly modeled and predicted bark allometry as a function of plant growth rate, bark benefit to survival, and tree size defensive benefit [25]. This past work predicted that positive allometry is favored by a convex tree size to fire deterrence relationship (decreasing marginal benefit to height with growth), and a concave bark thickness versus deterrence relationship (increasing marginal benefit of bark thickness with tree size). In contrast, rapid early growth, and a convex bark thickness versus deterrence relationship favored negative allometry. The authors' empirical test of the model assumed that habitats with infrequent but severe fire would create conditions favorable to positive allometry (thin sapling bark and accelerating investment with tree size) and that frequent low severity fire should favor negative allometry (thick sapling bark and decreasing investment with tree size). More recent work has supported these results by demonstrating that relative bark thickness as a measure of bark allocation varies with disturbance frequency and severity [26], [27].

Both Jackson et al. [25] and several more recent studies comparing bark thickness across many species [17], [18], [27] have focused on the survival traits of tree species that experience marked differences in fire regime (e.g. forest vs savanna species). Even within a single area and fire regime, however, multiple fire response strategies are possible: for example, non-sprouting fire recruiters and vigorous resprouters can co-occur within Mediterranean-climate shrublands [28], [29]. Furthermore, fire regimes can vary at small spatial scales [30], [31]. One unanswered question is the degree to which allometric patterns of allocation might allow local niche differentiation over relatively small spatial scales. Work on Florida oak communities has shown extensive niche differentiation among species occupying different parts of a subtle topographic gradient [26]. These Floridian oaks exhibit consistent differences in sapling bark thickness according to predicted fire regime with the thickest sapling bark in sandhill communities subject to frequent low severity fire and the thinnest bark in scrub communities subject to predictable, but severe, crown fire [26]. Past bark studies, however, have not examined if resprouting ability might shift optimal bark allocation.

Resprouting relies upon stored energy reserves and trees accumulate reserves in various storage organs. In disturbance prone systems, these organs are typically underground which protects them from the brunt of surface disturbances [13]. Root systems serve as a generic site of carbohydrate reserves which are then mobilized following a disturbance [32]. However, some species have specialized below ground organs (such as burls or lignotubers) that provide both a bud bank and carbohydrate storage. If bark investment is a defense against fire damage, investment in carbohydrate reserves may be an insurance in the event of fire.

We explored bark and carbohydrate allocation strategies across a suite of species that co-occur in a landscape that experiences a mixed fire regime [33], [34]. In these Chihuahuan desert Sky Island mountains, trees occur at mid to high elevations in communities with strikingly different fuel loads from grass-dominated savannas at the lowest or driest sites to closed canopy forest at the highest elevations and most mesic sites. Although oak species here do show moisture habitat preferences, there is extensive distribution overlap among species with as many as four oak species occurring within a few meters of one another (Schwilk, personal observation). We compared investment in bark and in root carbohydrates over ontogeny in eight common oaks in the Texas Trans-Pecos mountains. Our objective was to determine how these species, *Quercus emoryi*, *Q. gambelii*, *Q. gravesii*, *Q. grisea*, *Q. hypoleucoides*, *Q. muehlenbergii*, *Q. pungens* and *Q. rugosa*, invest in fire survival traits, including thick bark and resprouting. We measured bark thickness directly and used total nonstructural carbohydrate storage as a proxy for resprouting ability. We asked: how does investment in bark thickness and root carbohydrates change through the life of the tree and is there a tradeoff between the two alternative survival strategies?

We hypothesized that the timing of relative investment in bark can be predicted by local habitat preference that can influence fine scale fire return intervals and behavior [15]. These species can co-occur, but they do exhibit consistent soil moisture habitat preferences and different drought tolerances. At wetter sites, fire return intervals will be longer due to greater fuel and soil moisture. Fires are more severe at wet sites, however, when they do occur due to greater woody plant biomass and valley bottom fuel accumulation [35]. Recent fires in the Davis Mountains were highly variable in intensity [36] and this tended to vary with aspect and topography such that wetter sites with denser forest were more likely to experience crown fire (Schwilk, personal observation). Additionally, fire scar data indicated that mean fire intervals for upper topographic positions were 26 years and 34 years for valley bottoms in the Chisos and Davis Mountains [33]. We predict that species that inhabit wetter, denser sites will exhibit low initial bark investment and that relative investment will increase with tree size and age (allometric scaling coefficient, 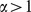). Drier sites with lower canopy cover and greater grass fuel loads will experience shorter fire return intervals and fire of lower intensity. Species preferring such sites will exhibit high initial bark investment and the rate of investment will decrease with tree size (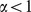). In more mesic environments, tree size works in conjunction with bark thickness; when trees are small bark thickness alone cannot save them from a fire, however when trees are tall then the combination of height and bark thickness contribute to enhanced survival [5], [24]. As an alternative to investing in thick bark, trees may invest in resprouting ability, represented by root nonstructural carbohydrates. We predicted that species in habitats which experience high severity fire will have a greater proportional investment in total nonstructural carbohydrates than species occurring in habitats with low severity fire. Due to resource limitation, there may a tradeoff in investment in carbohydrates and bark. Species that have increasing rates of investment in bark will have decreasing rates of proportional investment in carbohydrates. Conversely, species that have decreasing rates of investment in bark will have increasing investment in carbohydrates.

## Materials and Methods

### Ethics Statement

This field work was conducted under National Park permits BIBE-2010-SCI-0019 and GUMO-2010-SCI-0012 to Dylan Schwilk.

### Field Methods and Data Collection

We conducted this work in three mountains ranges in west Texas: the Chisos Mountains located in Big Bend National Park, the Davis Mountains in the Nature Conservancy Davis Mountains Preserve and the Guadalupe Mountains located in Guadalupe Mountains National Park. These ranges experience similar conditions and fire regimes and have similar potential microclimates [33], [34], [37], [38]. These mountain ranges are desert “Sky Islands” that support woodlands and forests at the higher elevations and Chihuahuan desert grasslands at lower elevations. We collected data from 249 trees in seven species at sites between 1,400 m and 2,400 m elevation.

The Guadalupe Mountains are the remnants of an ancient limestone reef. They have a semi-arid climate with an average yearly rainfall of 45 cm (National Park Service), and there are no perennial streams. The mean fire return interval is 22 years, with fires usually occurring in the spring [37]. The Davis and Chisos Mountains lay at the northern end of the Sierra Madre Oriental and are of igneous origins. Both have Chihuahuan grasslands at lower elevations segueing to piñon-juniper woodlands then up to conifer forest at the higher elevations [33]. These ranges have an arid climate, with precipitation occurring in winter and summer pulses. The mean annual precipitation in the Davis Mountains is 40 cm and 70 cm in the Chisos [37]. These ranges experience fire more commonly in the spring than other seasons. Fire scar records indicate that forested elevations in the Guadalupe Mountains have a mean fire return interval of 15 years [37], the Davis Mountains have a mean fire return interval of 11 years while the Chisos have a mean fire return interval of 36 years [33]. All three ranges have complex topography and multiple vegetation types allowing for heterogeneous fire regimes across the landscape.

The species of interest (*Quercus emoryi* Torr., *Q. gambelii* Nutt., *Q. gravesii* Sudw., *Q. grisea* Liebm., *Q. hypoleucoides* A. Camus., *Q. muehlenbergii* Engelm., *Q. pungens var. pungens* Liebm., *Q. rugosa* Nee.) were chosen based on reconnaissance by the authors and previous work by H. Poulos [33], [38]. Trees were identified using Powell [39] and Muller [40], with ambiguous individuals considered possible hybrids and subsequently excluded (disregarding Muller's strict advice [40]). General habitat preference information was recorded during sampling and combined with information on distribution, topography and soil moisture in Poulos and Camp [38] to characterize habitat preference as either “wet” or “dry.”

We selected individuals of each species for bark sampling by a stratifying across elevation and size classes. General start points for sampling were spread along an elevational gradient dictated by the lower and upper bounds of the species€ distribution in each range. First a species and size class was chosen: seedling (1 cm–10 cm), small (11 cm–20 cm), medium (21 cm–30 cm), large (30 cm–40 cm) and very large tree (41 cm +). The researcher randomly selected a point along a road or trail in the needed region and elevation, then selected a direction at random and walked up to 200 m in that direction. If the researcher passed within 10 m of tree of the given species and size, then it was sampled. If after 200 m no tree matching the criteria was encountered, a new combination of tree and size was selected and the process continued. An objective of at least 5 trees per size class per mountain range was set, excluding the “very large” category. Every effort was made to collect 30 trees per species per mountain range. However, several species were only found in one or two ranges. Total sample sizes were *Quercus emoryi*, n = 59; *Q. gambelii*, n = 37; *Q. gravesii*, n = 28; *Q. grisea*, n = 56; *Q. hypoleucoides*, n = 29; *Q. muehlenbergii*, n = 26; *Q. pungens*, n = 14; *Q. rugosa*, n = 6). Total nonstructural carbohydrates were assessed on a subset of 6–22 individuals of each species, randomly selected from the sampled individuals but stratified to capture at least one individual per size class.

**Figure 1:**
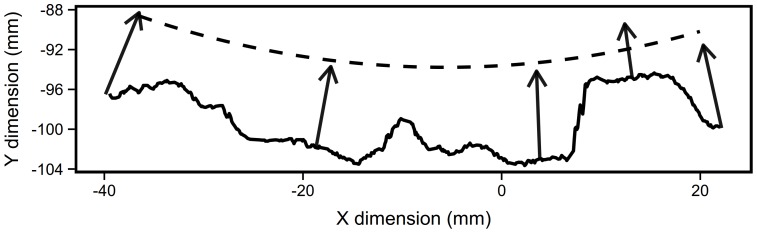
Example bark contour (*Q. emoryi*). Solid line shows the digitized outer bark contour created from the carpenter's molding gauge impression. Arrows indicate the bole depths drilled at five locations. The dashed line indicates the estimated inner bole surface fit using a smoothing spline in polar coordinate space. The unit are in mm with the origin at the estimated center of the bole.

Bark thickness was measured following the contour method of Adams and Jackson [15]. This minimally destructive method not only provides mean bark thickness it also allows for collection of the full distribution of bark thicknesses along a sample cross section. Once a tree was selected, the diameter was taken at 60 cm (hereafter referred to as D60), then an aspect (azimuth) at which to measure bark was randomly selected from the full 360 degrees. The outer bark profile was obtained by pressing a carpenter's molding gauge firmly against the bark surface; the profile was traced onto graphing paper (Fig. 1). Bark (including phloem) depth was taken at five points along the profile by drilling five holes with a doweling bit and then measuring the depth to xylem with a digital depth gauge. The location of the holes as well as the bark depths were recorded on the tracing of the bark profile.

### Bark Measurements

The area and the depths of the bark cross-section were determined using a process that started with scanning and digitizing the bark contour outline, the 5 drill point locations and corresponding measured bark depths using ImageJ [41]. The perimeter scan was saved as X-Y coordinates and then analyzed with a custom program written in R [42] (code in Text S1 and available at http://www.schwilk.org/research/data.html). The algorithm comprised four steps.

1. We estimated the location of the center of the bole from the diameter tape measurement and the 95th quantile most outer bark contour points which would represent the portion of the contour contacted by the diameter tape. This step was iterative: establishing the relative position of the bark contour points required an initial estimate of the bole center using the drill point locations. The initial center estimate was the average intersections of the five circles of the same radius centered on each of those points. The final bole center was estimated as the average of the intersections of the circles described by the measured tree radius centered on each estimated tape contact point (the local bark thickness maxima).
2. All coordinates were transformed to polar coordinates with the origin at the previously estimated bole center. Then the five drill depths were used to establish five inner cambium positions along the rays passing through each drill point.
3. A smoothing spline was applied on these inner cambium points to estimate the curve of the inner bark boundary. This curve was fit in polar coordinate space to maintain circularity as the default geometry but to allow for non-circular tree boles. We found that a smoothing parameter of 0.7 in R's smooth.spline function [42] best fit our visual estimation of inner bole shape for test tree stumps for which we could view entire cross sections.
4. With both an inner bole and outer bark contour estimated, the program returned an ordered vector of bark depths along the arc circumscribed by the traced contour (Fig. 1). For seedlings and saplings a small area of bark was shaved from the tree and bagged to maintain moisture, then bark thickness was measured under a dissecting scope.

After the bark profiles were digitized, the mean bark depth was found for each species at three sizes representing the range of trees sampled. We predicted bark thickness and critical cambium survival time at two diameters for comparison across species. The smallest size (D60 = 10 cm) represents small adult trees (around the 12th quantile over all individuals), the medium size (D60 = 20 cm) was near the median size across all individuals and the largest size (D60 = 30 cm) represents the 80th quantile D60 across individuals and species. Following previous work [20], the mean bark depth for each size class was converted to a critical time (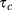) using the equation for one dimensional heat flow through bark [20].
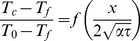
(1)Where 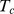 is the critical temperature for cambium mortality (°C), 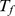 is the assumed flame temperature (°C), 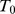 is the ambient temperature of the bark (°C), 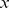 is bark thickness (cm), 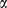 is the bark thermal diffusivity (cm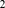/min), 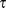 is time (min) and 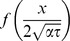 is the Gaussian integral. The left hand side of the equation is not very sensitive to realistic ranges of temperatures. If we assume a lethal temperature (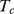), then, by referring to a table of the Gaussian integral and by rearrangement, we can solve the equation for the critical time needed for cambial mortality, 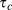. Assuming realistic values for ambient temperature, flame temperature and bark thermal diffusivity, 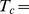 60°C [20], [43], 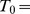 20°C, 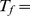 500°C, 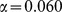 cm^2^/min [20], [44] then:



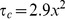
(2)This critical time, 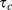 predicts the time of fire exposure at 500°C that would result in cambium death. Other researchers have found variation in thermal diffusivity of bark and some variation in this relationship [45], but the power law of critical time rising as the square of bark has proven general.

### Root Carbohydrate Measurements

We measured total nonstructural root carbohydrates on a subset of ten individuals per species per mountain range selected to capture the range of sizes present by selecting at least 3 trees per size class per species. In species with limited geographic ranges the minimum collected was ten, except for *Q. rugosa* of which only six trees were found (*Quercus emoryi*, n = 22; *Q. gambelii*, n = 20; *Q. gravesii*, n = 11; *Q. grisea*, n = 24; *Q. hypoleucoides*, n = 10; *Q. muehlenbergii*, n = 13; *Q. pungens*, n = 12; *Q. rugosa*, n = 6).

All root samples were collected between May and June 2011, which was after initial spring leaf flush and before secondary shoot development to indicate yearly minimum carbohydrate storage. Samples were collected from the tap root of each tree and kept on ice then temporarily moved to a 4°C freezer. Upon returning to the lab, root material was stored in a drying oven at 50°C until time of analysis. We assessed total nonstructural carbohydrates using a rapid method [46]; in which dried material was ground for 60 seconds in a mill resulting in a mix of fine powder and coarse debris. Samples were soaked in a methanol-chloroform-water combination for one and a half hours, centrifuging and pouring the liquid component off at 30 minute intervals. Solid material was air dried overnight and the liquid was stored in a refrigerator overnight. The following day the solids were mixed with perchloric acid for an hour resulting in a fluid containing digested sugars; a small portion was then mixed with water, phenol and sulfuric acid. The resulting solution was read in a spectrophotometer at 490 nm. The same process was conducted, without the addition of perchloric acid, for the refrigerated portion. The absorbance readings for the liquid and solid portions of each sample were adjusted according to a glucose blank and then combined to give percent total nonstructural carbohydrates.

### Analysis

Bark allometry curves were developed for each of the species by using the smatr package in R to calculate the standard major axis regression (SMA) [47], [48] between diameter at 60 cm and bark depth of log transformed data. The allometric coefficients (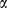) of each species were compared to the isometric coefficient (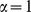) and to one another using the multiple comparisons functions in the smatr package. Because SMA analysis indicated that slopes differed among species, an overall SMA test of elevation differences was not justified. Instead, in order to test the effect of soil moisture habitat on slopes and intercepts (sapling bark thickness), we used a nested linear model with species nested within habitat type. For carbohydrate storage, diameter at 60 cm, total nonstructural carbohydrate concentration and date of lab work were compared using a linear mixed effects model in R with the nlme package [49], [50]. Diameter did not predict carbohydrate levels, therefore we ran a single factor ANOVA across species followed by a Tukey Honest Significant Differences post-hoc test to compare carbohydrates across species. Then bark allometric coefficients were compared against the patterns of total nonstructural carbohydrate storage.

## Results

Due to its restricted range, *Q. rugosa* was excluded from analysis of bark thickness calculations. Our bark allometry data, therefore, included seven *Quercus* species. Based on Poulos et al. [38] and observations of sampled trees, *Q. emoryi*, *Q. gambelii*, *Q. grisea* and *Q. pungens* prefer more xeric habitats and *Q. gravesii*, *Q. hypoleucoides* and *Q. muehlenbergii* prefer mesic habitats (hereafter referred to as “dry” and “wet” habitat preferences, Table 1). Among the dry habitat types *Q. emoryi* and *Q. grisea* were distributed in savanna and open canopy woodland including areas near draws; while *Q. pungens* was typically found in low elevation desert grasslands growing as a less than a meter tall multi-stemmed shrub. The wet habitat group included species found most often in higher elevation canyons, *Q. gravesii* as well as lower elevation species that preferred canyons and drainages, *Q. hypoleucoides* and *Q. muehlenbergii*.

**Table 1:** Eight oak species studied.

Species	N	Habitat Preference	Section	Mountain Ranges
*Q. emoryi*	59	Dry	Red	Chisos, Davis
*Q. gambelii*	37	Dry	White	Chisos, Davis, Guadalupe
*Q. gravesii*	28	Wet	Red	Chisos, Davis
*Q. grisea*	56	Dry	White	Chisos, Davis, Guadalupe
*Q. pungens*	29	Dry	White	Chisos, Guadalupe
*Q. hypoleucoides*	26	Wet	Red	Chisos, Davis
*Q. muehlenbergii*	14	Wet	White	Chisos, Guadalupe
*Q. rugosa*	6	Wet	White	Chisos, Davis

Table shows number of individuals sampled for bark thickness (N) and soil moisture habitat preference. “Section” refers to the major taxonomic groups within *Quercus*, of which two are represented by our species. White = section *Quercus* or *Leucobalanus*. Red = section *Lobatae* or *Erythrobalanus* [65]. “Mountain Ranges” indicates from which ranges each species was collected.

**Figure 2:**
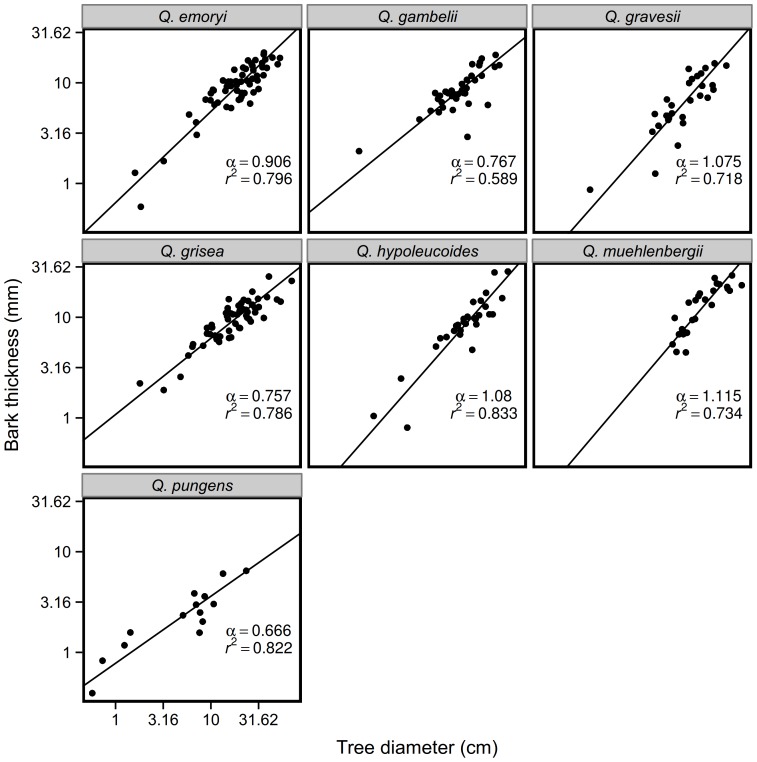
Allometric relationships between tree diameter and mean bark thickness across species. Lines and coefficients represent standardized major axis regression [47].

**Figure 3:**
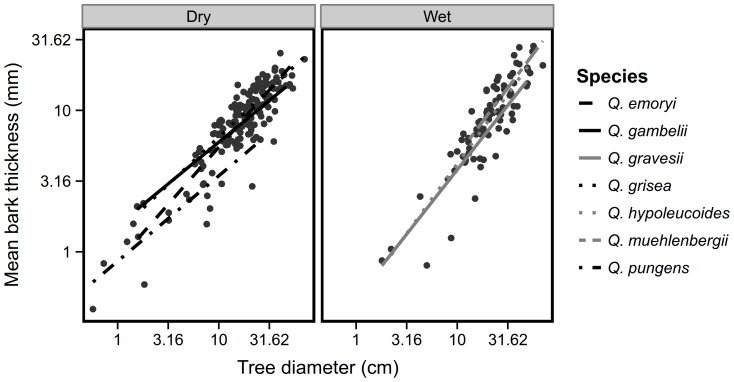
Allometric relationships between tree diameter and mean bark thickness across species by habitat preference. The species preferring dry sites have shallower slopes (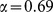 ) than the species inhabiting wet sites (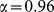). A linear mixed ANCOVA model with trees nested within species (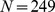, groups = 7, 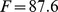 on 13 and 235 d.f., 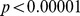, model 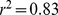) indicates significantly different slopes (at p = 0.003).

Mean bark depths ranged from 0.4 mm to 30.0 mm with the majority falling between 6.2 mm and 12.9 mm (25th and 75th quantiles). Bark allometry ranged from negative (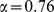) in *Q. grisea* to weakly positive in *Q. muehlenbergii* (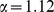). Allometric slopes were significantly lower than one in *Q. grisea*, *Q. gambelii*, and *Q. pungens* (all 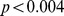), while the others had isometric allometries with slopes not distinguishable from one (Fig. 2). A mixed linear model showed that slopes differed significantly among habitat groups: the species preferring dry sites had shallower slopes (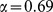 ) than the species inhabiting wet sites (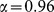) (habitat effect on slope 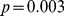, Fig. 3). For example, *Q. grisea* invests early in bark and reduces investment through time. In contrast, *Q. hypoleucoides* has thin bark in young stems and it accelerates investment over time resulting in among the thickest bark when mature. *Q. pungens* had thinner bark across all tree diameters than did the other species (Fig. 2–3).

Critical times for fire survival (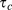, Equations 1–2) varied greatly across species. For smaller trees (D60 = 10 cm), estimated mean survival times ranged from around 20 seconds in thin-barked *Q. pungens* to over a minute for *Q. grisea*. For mid-sized trees (D60 = 20 cm), 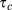 was greater than three minutes for all species except *Q. gravesii*, *Q. hypoleucoides* and *Q. pungens* (Table 2). For 30 cm diameter trees, predicted critical times were greater than three minutes for all species except *Q. pungens* which did not reach this size. The overall critical times do not change substantially if we use the empirical relationships between bark thickness and critical time reported by Lawes et al. for Australian savanna [45] rather than the Peterson and Ryan equation developed for conifers [20] (results not shown).

**Table 2:** Predicted time a tree can survive a fire at 500°C without fatal damage by tree diameter at 60 cm height.

Species	D60 = 10 cm			D60 = 20 cm			D60 = 30 cm		
	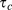	lwr	upr	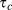	lwr	upr	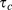	lwr	upr
*Q. emoryi*	0∶53	0∶16	2∶54	2∶41	0∶49	8∶50	5∶10	1∶34	17∶06
*Q. gambelii*	1∶02	0∶18	3∶27	2∶20	0∶42	7∶43	3∶45	1∶07	12∶36
*Q. gravesii*	0∶25	0∶07	1∶25	1∶29	0∶27	4∶57	3∶06	0∶55	10∶30
*Q. grisea*	1∶13	0∶22	4∶01	3∶04	0∶56	10∶07	5∶18	1∶36	17∶34
*Q. hypoleucoides*	0∶30	0∶09	1∶43	1∶60	0∶36	6∶38	4∶26	1∶19	14∶51
*Q. muehlenbergii*	0∶40	0∶11	2∶26	2∶31	0∶45	8∶26	5∶27	1∶38	18∶12
*Q. pungens*	0∶21	0∶06	1∶12	0∶48	0∶13	2∶54	-	-	-

Mean predicted critical time 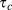 (min:sec) is shown for three different basal diameters, 10 cm, 20 cm and 30 cm (Equation 2). Lower and upper 95% confidence intervals are also shown based on linear model predicting (log) bark thickness from (log) basal diameter and species.

Multiple samples of root tissue (2–4) for carbohydrate analyses from each tree were measured after grinding and mixing material in order to gauge the precision of the lab method. The date the lab work was performed influenced carbohydrates measured: earlier dates showed higher concentrations than did the later dates, indicating some loss in detectable carbohydrates during storage. Therefore, we assumed that the earliest dates were most accurate and chose the earliest measurement for each individual tree and discarded the others (all measurements were within two months of collection).

**Figure 4:**
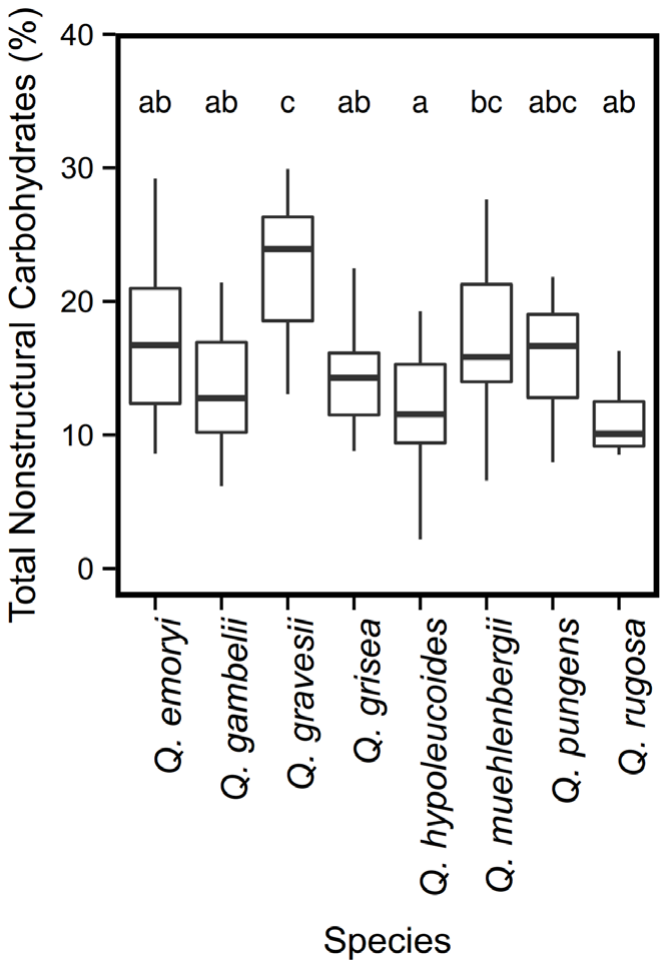
Mean percentage total non-structural carbohydrates by species. ANOVA results indicate significant effect of species (N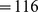, df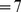, F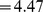, p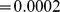). Bold horizontal bar represents median value; boxes include 1st and 3rd quartiles and lines extend to data range. Letters are assigned groups according to Tukey HSD post-hoc analysis: species that do not share letters have significantly different mean carbohydrates (p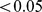).

Total nonstructural carbohydrate concentrations did not vary significantly with tree size (D60). ANOVA results indicate significant effect of species (N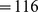, df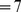, F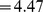, p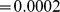). Overall, however, species had similar high levels of total nonstructural carbohydrate storage with one exception; *Q. gravesii* had significantly higher mean amounts than *Q. emoryi*, *Q. gambelii*, *Q. grisea*, *Q. hypoleucoides* (Fig. 4, 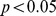 according to Tukey HSD).

## Discussion

Among the low-statured oaks that inhabit the west Texas mountains, survival following fire often depends upon some degree of resprouting because canopy tissue loss is common. Even larger trees experiencing relatively low severity fire may lose most of their leaves to heat damage and resprout from apical, axillary, and epicormic buds (Schwilk, personal observation). Resprouting response depends both on a species' ability to resprout and upon the intensity and frequency of the disturbance regime. The type of resprouting is also influenced by disturbance frequency. Aerial resprouting most often occurs with low severity disturbance (although *Eucalyptus* and some oaks can epicormically resprout following very severe fire, [51], [52]. Basal resprouting occurs after high severity disturbance [4], [53]–[55]. For example, Florida scrub oak species under a severe fire regime both resprouted basally and from rhizomes [26]. The investment in buds and storing nutrients for future use comes at a cost to other investments such as defense structure, current growth or future reproduction [3], [13]. In a study of four oak species and two pine species a negative relationship between post-fire resprouting ability and fire resistance (thick bark) was found; implying a possible tradeoff between the two alternate strategies [56]. A study of oaks in Florida found that saplings possessing thick bark occurred in sandhill habitats that experienced frequent low intensity ground fires, whereas species in habitats that experienced severe fires did not invest in thick bark [26].

Investment in bark changes over the lifetime of a tree. The species we studied exhibit a range of proportional investment in bark patterns through ontogeny: early then decreasing investment (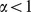), late investment (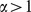) and constant investment (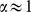). Taking habitat preference and associated fire regime into account, our results generally followed the predictions of Jackson et al. [25] and the empirical work by Cavender-Bares et al. [26]: dry site species had shallower allometric slopes than did wet site species (Fig. 3). The dry site species *Q. emoryi* and *Q. grisea* are likely to experience frequent low severity fires both had allometric coefficients significantly less than one. In contrast, species which prefer wetter sites and therefore likely to experience infrequent but high severity fires such as *Q. hypoleucoides*, *Q. gravesii* and *Q. muehlenbergii* had thin bark early with increasing rates of investment in bark. One species deserves some discussion: Although *Q. gambelii* was categorized as a dry site species, it does inhabit higher elevation sites which are sometimes wetter in addition to dry slopes and it may experience a range of fire return intervals. Finally, *Q. pungens*, which is both the lowest elevation species in the study and the species most restricted to a shrub-like growth form, had thinner bark across all diameters than the other species. This species does not appear to be investing in bark as a fire defense trait at all and instead relies completely upon resprouting for persistence through fire which is consistent with its growth form.

Bark allometry indicates relative investment cost, but the benefit of increased bark in increased critical cambium survival time (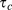) rises as the square of bark thickness [20]. At 10 cm, the dry site tree species, *Q. grisea* and *Q. gambelii* have the longest critical times, over a minute, and *Q. emoryi* is a close third. By the time they have reached this size, the dry site species have more likely experienced a fire than either canyon or wet site specialists [34]. The species found in wet canyons (*Q. gravesii*, *Q. muehlenbergii* and *Q. hypoleucoides*) can survive less than 40 seconds of ground fire at 500°C at 10 cm. These species may be able to grow to 10 cm size with a low probability of being exposed to fire; as fire return intervals for wet sites are typically lower than in dry sites [31] and growing in wet canyons can provide a refuge from fire. Once trees have reached a large size (D60 = 40 cm), the mean 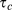 exceeds five minutes and the lower 95% confidence bound exceeds a 1.5 minutes for all but *Q. pungens*, which does not reach this size. Our bark measurements allow gross comparisons across species useful for characterizing investment strategies, but more realistic simulations of bark's insulating effects are possible. For example, recent simulation tools allow for detailed quantification of stem heating and tree mortality that incorporates information on bark thickness and structure (outer and inner bark), and starting bark moisture [57].

In addition to thick bark, growth form and size contribute to fire resistance [25]. We do not have direct information on growth rates for these species, but work on wet and dry site specialists in the nearby Sierra del Carmen, Mexico, demonstrated that a wet site species had faster leaf addition and height growth while the dry site species had greater rates of root collar diameter growth and a higher root:shoot ratio [58]. Furthermore, these trees do differ in growth form. *Q. gravesii* and *Q. muehlenbergii* were single-trunked trees and generally taller than the often multi-stemmed trees *Q. gambelii*, *Q. grisea*,*Q. emoryi* and *Q. pungens*. *Q. hypoleucoides* was found as both a multi-stemmed shrub and as single-trunked individuals suggesting that it may switch between survival strategies, possibly resprouting more vigorously when small or shrub like. *Q. hypoleucoides* shifts to greater bark investment later in ontogeny (Fig. 2, which is consistent with the shift in growth form. Unlike the rest, *Q. pungens* was exclusively found as a multi-stemmed shrub with no single stem reaching 25 cm in diameter. This, along with the very thin bark across all diameters in this species suggest it may rely more heavily on resprouting than on above-ground survival during fire.

Resprouting relies upon a bud bank, bud protection and carbohydrate storage [13]. The oaks included in this study are all post-fire resprouters (Poulos and Schwilk, personal observations), which is consistent with the results reported here which show relatively high root carbohydrate investment across all species, higher than values reported for other vigorous post-fire resprouters in shrub systems [9], [59], [60], although studies on oaks and other deciduous forest trees have reported similar values [61]–[64]. Our root carbohydrate results for *Q. gambelii* are very similar to values reported for the species in previous work [61]. Root carbohydrates, however did not vary with tree size. Furthermore, contrary to our prediction, species did not fall into groups related to habitat preference or to growth form. Although there was no relationship between habitat preference and total nonstructural carbohydrate concentration, *Q. gravesii* had a significantly higher mean carbohydrate concentration than *Q. emoryi*, *Q. gambelii*, *Q. grisea*, and *Q. hypoleucoides*. This difference remains unexplained, however, because *Q. gravesii* is not a noticeably more vigorous resprouter than these other species (Schwilk, personal observation). This difference deserves greater attention and a rigorous measurement of resprouting vigor following fire is warranted.

We found a range of bark investment strategies and this variation suggests a trade-off between early investment in bark versus investment in growth. On the other hand, we found no support for a trade-off between bark and carbohydrate investment. Although we do not have actual information on growth rates, 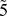-year-old post-fire resprouts of Q. emoryi were larger and taller than same-age resprouts of *Q. grisea* in the Davis Mountains, which is consistent with a trade-off between early bark investment and growth rate (Schwilk, personal observation). Despite clear differences in bark allocation across species, we do not see any related differences in carbohydrate storage, at least on a percent dry root tissue basis. A decreased investment in bark defense should result in greater reliance on basal resprouting and therefore on stored carbohydrate reserves. On the other hand, Cavender-Bares et al. (2004) [26] found that rhizome resprouting ability was high in both thin-barked Floridian oaks that experience severe fire as well as in thick-barked sandhill species that experience frequent low-severity fire. It is possible that these Texas species do differ in total below-ground carbon storage, but do so through differences in total root mass rather than in differing root carbohydrate concentrations. Alternatively, it is possible these are orthogonal strategies, with species investing in bark and carbohydrate storage independently. Other traits, such as mature tree height could also play an important role as a survival trait [25]. Although all the oaks in this system are of relatively low stature, the tallest individuals of all species except for *Q. pungens* may be able to escape extensive canopy tissue damage during low intensity fires.

This study demonstrates the potential importance of bark allometry for niche differentiation. These species show consistent differences in bark allocation strategies according to soil moisture preference and presumed local fire regime. Although the clearest benefit of thick bark in this system is in protection of the cambium against fire damage, bark has multiple functions including providing a barrier to insect and fungal pathogens, and perhaps preventing water loss. Bark functions other than insulation, however have received little or no empirical testing and are generally speculative. Our ongoing work with these species suggests that soil moisture habitat preference, treated here as a simple dichotomy, represents continuous variation in xylem vulnerability to drought-induced cavitation. For example, of these species, *Q. grisea* has the shallowest bark allometry and is the most resistant to drought-induced embolism (it does not reach 50% loss of xylem conductivity until water potential drops to nearly -3 MPa, Schwilk and Lackey unpublished data) while the species with the steepest bark allometries, *Q. gravesii* and *Q. hypoleucoides*, are the least resistant (50% conductivity loss at water potentials around -1 MPa, Schwilk and Lackey, unpublished data). Unlike previous work comparing forest and savanna species, this study demonstrates that differences in bark allocation presumably due to fire response strategy can vary at very small spatial scales. Although these species have differing soil moisture and elevation preferences, there are multiple sites where up to four of these oak species can co-occur within 50 m of one another. In these forests subject to a mixed fire regime, spatial and temporal variability in fire regime supports variability in fire survival strategies.

## Supporting Information

Text S1 **R code to calculate bark thickness distributions from a digitized contour.**
(R) Click here for additional data file.
